# Slum Upgrading and Health Equity

**DOI:** 10.3390/ijerph14040342

**Published:** 2017-03-24

**Authors:** Jason Corburn, Alice Sverdlik

**Affiliations:** Department of City and Regional Planning & School of Public Health, University of California, Berkeley, CA 94720, USA; sverdlik@berkeley.edu

**Keywords:** slums, health equity, slum upgrading, social determinants of health, climate change adaptation, housing, participation, sustainable development goals, health in all policies

## Abstract

Informal settlement upgrading is widely recognized for enhancing shelter and promoting economic development, yet its potential to improve health equity is usually overlooked. Almost one in seven people on the planet are expected to reside in urban informal settlements, or slums, by 2030. Slum upgrading is the process of delivering place-based environmental and social improvements to the urban poor, including land tenure, housing, infrastructure, employment, health services and political and social inclusion. The processes and products of slum upgrading can address multiple environmental determinants of health. This paper reviewed urban slum upgrading evaluations from cities across Asia, Africa and Latin America and found that few captured the multiple health benefits of upgrading. With the Sustainable Development Goals (SDGs) focused on improving well-being for billions of city-dwellers, slum upgrading should be viewed as a key strategy to promote health, equitable development and reduce climate change vulnerabilities. We conclude with suggestions for how slum upgrading might more explicitly capture its health benefits, such as through the use of health impact assessment (HIA) and adopting an urban health in all policies (HiAP) framework. Urban slum upgrading must be more explicitly designed, implemented and evaluated to capture its multiple global environmental health benefits.

## 1. Introduction

Few urban initiatives have greater potential to promote health equity and to advance the well-being of poor households in the Global South than participatory, multi-objective slum upgrading. Urban informal settlements are communities that are highly diverse and have a range of locally-specific names (such as barrios, bustees, mjondolo, or favelas). Here we use the terms “slums” and “informal settlements” to denote largely self-built urban communities, which are rarely recognized officially and typically are denied life-supporting services and infrastructure. Not only can upgrading these settlements improve health outcomes via enhanced access to shelter, water, or clean energy, but it can also advance the 2030 Agenda for Sustainable Development. The 2030 Agenda has established 17 interrelated Sustainable Development Goals (SDGs), which will require implementing multi-sectoral strategies to support health and well-being and include explicit reference to slum upgrading. Yet, as we will highlight, upgrading projects are rarely designed to explicitly improve nor are they evaluated for how well they have positively shaped the social determinants of slum health.

The social determinants of health (SDOH) are factors outside medical care that shape health outcomes, such as safe housing, food access, political and gender rights, education and employment status [1]. In urban informal settlements, residents are often burdened with multiple and overlapping challenges that can undermine the SDOH, from entrenched poverty, to overcrowded shelter, to inadequate infrastructure and tenure insecurity, all of which can combine to contribute to increased risk of exposures to environmental pathogens that increase infectious and non-communicable diseases in urban poor areas [2,3]. Slum-dwellers also experience spatial, political, and economic exclusion when compared to wealthier urban residents, and these too are recognized as powerful determinants of who gets sick, suffers more and dies early [4].

In this review article, we analyzed slum upgrading projects and published evaluations of these projects or policies for whether and how they captured the influence of upgrading on the SDOH [5]. We analyzed completed slum upgrading projects from a dozen different countries and performed content analyses to determine if these projects included explicit measures of any SDOH and/or specific disease outcomes. We focused our review on upgrading projects and policies that took an “integrated” (i.e., multi-sectoral) approach to upgrading since we determined that these aimed to influence the SDOH through interventions focused on more than one physical, social and/or political issue facing the urban poor [6,7]. We also limited our review to slum upgrading interventions where we could identify at least two published evaluation reports. By incorporating a wide range of peer-reviewed and grey literature, we aimed to distinguish our study from past research that only reviewed the health outcomes, but not necessarily the broad determinants, of slum upgrading projects utilizing randomized studies [8]. As discussed in more detail below, we found that a majority of slum upgrading projects rarely measured how upgrading projects affected health outcomes or social determinants. We suggest that this represents a lost opportunity for health promotion and conclude with some suggestions for how slum upgrading might more explicitly promote the SDOH amongst the urban poor in the Global South [9].

## 2. Defining Slums, Upgrading, and Their Relationships to Health Equity

Slum-dwellers increasingly face a “triple threat” of infectious diseases, non-communicable conditions (e.g., diabetes, cardiovascular disease, and mental illness), and injuries due to violence or road traffic accidents. Table 1 offers some definitional characteristics of slums and associated environmental health issues. However, there is no single adequate definition of slums, since they are often heterogeneous in terms of housing types, poverty prevalence, tenure (in)security, levels of infrastructure and service provision, environmental hazards and health risks [10].

Similarly, there is no single definition of “slum upgrading”, as projects and policies differ based on region of the world, political and development histories, and other factors [11]. Beginning in 1972, the World Bank launched urban upgrading projects to improve services, infrastructure and housing in hopes of reducing poverty and meeting basic needs [12]. Yet governments often persisted in slum clearance and neglected to maintain what little infrastructure had been upgraded. By the 1980s–1990s, international development agencies shifted their approach to slum upgrading; rather than construct housing and infrastructure, governments acted as “enablers” by offering financing for mostly non-governmental organizations’ projects, which largely focused on housing [13]. By the early 2000s another shift occurred and a greater emphasis was placed on in-situ upgrading, limiting slum clearance and launching policies that aimed to integrate the urban poor into the larger fabric of growing metropolitan economies [6,13].

As used here, “slum upgrading” denotes initiatives seeking to improve housing quality, infrastructure provision, social services, livelihoods, and official recognition for residents. Slum upgrading is also a process that meaningfully includes the urban poor in project and policy design and implementation, aims to integrate slums into the larger fabric of the city, is attentive to emerging challenges of climate change adaptation, and is often incremental—meaning that residents remain in place while improvements occur to prevent displacement (cf.: http://www.citiesalliance.org/About-slum-upgrading#Why_is_slum_upgrading_important). Yet, the scope of upgrading can vary from small-scale sector-specific projects (i.e., water-taps, paved roads, street lighting) to comprehensive housing and infrastructure projects (i.e., piped water and sewers into improved housing) to integrated projects that combine built-environment interventions with social programs and political empowerment [13,14]. Many upgrading programs have also focused on providing residents with legal rights to land and housing tenure [15]. Importantly, slum upgrading requires state recognition of the urban poor, their settlements, and the need to deliver basic services. By granting official legitimacy to slum-dwellers and their “right to remain”, upgrading represents an important alternative to slum removal and may be a process that in itself may offer health benefits [8]. However, as we explore below, upgrading projects are rarely evaluated on their ability to positively influence the social determinants of health.

## 3. Methods: Review of Urban Slum Upgrading Projects

Our analysis of slum upgrading evaluations incorporated both grey literature and published peer-reviewed studies, with a focus on integrated interventions. We started with a scan of published literature between 1997 and 2016 through on-line databases using general search terms, including “informal settlement”, “slum”, “upgrading” and “health”. Based on this review, we found 182 possible evaluations but narrowed them down to thirty-two by selecting those that had at least one additional published report on the project. We also narrowed our search by focusing upon “integrated” or multi-sectoral interventions, which aimed to address physical, social and urban governance concerns in informal settlements [26,27]. We focused our search on multi-sectoral interventions because we hypothesized that these might offer reviews and/or evaluations of several environmental and social impacts. Further, global slum upgrading programs, such as UN-Habitat’s Participatory Slum Upgrading Program, call for integrated or “blended” projects that aim to achieve multiple objectives [28]. We also limited our search to find projects that might act as examples of the type of cross-sector interventions called for under the Sustainable Development Goals [29]. We utilized the WHO definition of SDOH to sharpen our search terms and refined our findings into three categories under which upgrading initiatives may influence health in informal settlements: national and municipal policies, structural determinants, and living and work conditions [1,9]. By “national and municipal policies”, we mean any legislation or government-sponsored initiatives that may positively influence the SDOH, including a slum upgrading policy or financing scheme. This category thus underscores that governmental decisions and priorities—often outside the medical and public health sectors—are key drivers of population health. By “structural determinants”, we focus on the specific population drivers of health inequities frequently identified in the SDOH literature, including income and wealth, educational access, employment, early childhood development, food security, and social exclusion. By “living and working conditions”, we focus on the physical characteristics of neighborhoods and urban places, such as access to quality and affordable housing, water, sanitation, electricity, transport, etc.

We selected nineteen interventions from Latin American, African and Asian cities that met our screening criteria; while not exhaustive, this list is meant to illustrate the range of slum upgrading projects globally and the potential of participatory, integrated projects for improving human health. We summarize select characteristics of each slum upgrading project and whether and how they were evaluated for impacts on human health in Table 2.

## 4. Findings: Slum Upgrading Projects and the Determinants of Health

Of our nineteen selected projects, thirteen evaluations directly discussed potential SDOH, such as reduced water collection times; enhanced community pride and social cohesion; greater levels of micro-savings, loans, or economic stability; and increased women’s empowerment. Community participation—residents’ and/or community-based organizations’ involvement in the project—was present in all but four of the upgrading evaluations we reviewed. However, these issues were rarely discussed as having potential benefits for population health. Eleven of the reviewed projects explicitly mentioned health impacts, using self-reported data or state collected data such as cause of death. Only three slum upgrading projects were evaluated for their influence on specific disease outcomes. In sum, our scan revealed the following:

- Health outcome measures were very limited and tended to focus on childhood mortality or morbidity (particularly due to diarrheal or other communicable illnesses);
- Economic impacts were frequently evaluated, but few studies discussed how poverty and/or income influenced the health status of slum-dwellers;
- Infrastructure improvements were measured through such variables as the number of new water-points, sanitary infrastructure or unit costs, but rarely analyzed how infrastructure might positively influence social or economic opportunities, safety, or reduce gender inequities;
- Resident participation, including through micro-savings, was frequently mentioned, but none of the reports attempted to link participation to potential positive or adverse human health impacts, the latter being social stigma and hypertension [69]; and,
- There was no consistent time frame for when evaluations took place and this varied from immediately after a project was completed to, on average, 18 months after completion.

### 4.1. National/Municipal Policies for the Urban Poor

We found that slum upgrading projects are typically place-specific, but more rarely part of ongoing national or municipal policy priorities. However, at least one-third of the projects included in this review had established national and/or municipal policies to support slum upgrading, including in Brazil, Colombia, Mexico, Nicaragua, India, Thailand and Kenya. In Thailand, the Baan Mankong program is supported by the Community Organization Development Institute (CODI), a national governmental agency that provides housing loans, infrastructure subsidies, and ongoing support to cities and communities to collaborate in slum upgrading projects [15]. Favela Bairro, an intervention of the Rio de Janeiro municipality with national and international fiscal support, similarly included projects focused on improving physical infrastructure and developing new social programs in favelas [49,70].

Although varying in scale and institutional arrangements, successful initiatives were often state-community partnerships that originated with state recognition of the rights and legitimacy of slum-dwellers. For instance, PRIMED in Colombia, Baan Mankong, and Favela Bairro were all large, state-sponsored slum upgrading initiatives where the urban poor were recognized as having rights and requiring a range of state services [15,47,50]. Having a supportive state policy framework can help to avoid boutique, one-off slum upgrading projects, which may have only limited long-term impacts upon population health. Central governments can provide sustained political and fiscal support for complex upgrading projects; municipal governments have also proved important in ensuring utilities and other service providers meet the needs of slum-dwellers [6].

### 4.2. Structural Conditions for Health

Many of our selected upgrading interventions aimed to address multiple structural inequalities, with significant potential to improve residents’ economic status and reduce gender inequities. In Manila, slum-dwellers receiving piped water under the Zonal Improvement Program reported a reduction in household water expenditures; the project also eliminated three to four hours per day of waiting time for water and as many as 72% of beneficiaries reallocated their time to income-generating activities [41]. Following Ahmedabad’s Slum Networking Project (SNP), Butala et al. [32] (p. 939) found an 18% decrease in the fraction of slum-dwellers’ insurance claims due to waterborne illness in an average year. The Neighborhood Upgrading and Shelter project in Indonesia improved water, sanitation, roads, and electricity for nearly three million people and resulted in avoided health care costs of $11 m annually [43]. During a community-led upgrading project in Huruma, Nairobi, slum-dwellers have formed micro-savings groups that build social and economic capital, and the initiative resulted in improvements in housing, infrastructure and political recognition [61].

Furthermore, after slum upgrading projects in Visakhaptnam, Indore, and Vijaywada, India, an evaluation noted that women particularly benefited from the enhanced nighttime security, improved water provision, and public lighting [31]. Similarly, an evaluation of Ahmedabad’s SNP found gender-equitable benefits from improving women’s access to credit and household electricity [34]. In this upgrading project, the electricity utility issued meters and monthly bills to female-headed households, providing these women with de facto tenure security since the utility and state had no interest in evicting rate-paying customers.

### 4.3. Living and Working Conditions

Evaluations typically mentioned the projects’ direct influence upon neighborhood living conditions and occasionally linked these to health outcomes or determinants, such as fear and safety. For example, projects in Cali, Bogotá, and Medellín, Colombia, all found some changes in residents’ attitudes towards the police and improved perceptions of safety, but did not measure cortisol, anxiety or other risk factors for mental illness [59]. Similarly, residents of favelas in Salvador, Brazil, reported a decline in fear of crime after upgrading [53]. Evaluations of Favela Real in Paraisópolis, Sao Paulo, found that improved electricity infrastructure offered residents an address and opportunity to gain state benefits that they had been previously denied [51]. Meanwhile, after upgrading Cape Town’s informal settlement of Imizamo Yethu, beneficiaries felt greater community pride and social inclusion, but shack-dwellers who did not receive upgraded houses reported increased stress, stigma, and social exclusion [63]. In India, the Philippines, and Pakistan, slum-dwellers have planned, financed, and implemented sanitation initiatives with significant positive influence on environmental determinants of health [32,41,46]. For instance, in Karachi, community-led sanitation interventions under the Orangi Pilot Project have been linked to decreases in infant mortality and enhanced government recognition of slum-dwellers [46]. In Ahmedabad, the Mahila Housing Trust (MHT), the housing cooperative of the Self-Employed Women’s Association (SEWA), led a sanitation project that reduced by 50% the likelihood of a resident reporting a waterborne illness [32].

## 5. Discussion: Integrating Health Equity into Urban Slum Upgrading

Including health and social determinants criteria into slum upgrading projects is clearly challenging. Our scan of the literature found a limited number of integrated evaluations, and some reasons might be that there are few incentives from donors and national governments for such studies, that there are methodological challenges that make impact evaluations challenging, and resources may not exist after project implementation to conduct detailed studies of often highly mobile urban populations [71]. Further, as Cameroon et al. note from a systematic review of impact evaluations between 1981 and 2014, very little impact evaluation evidence exists of interventions in urban development, including informal settlements [71]. The World Bank’s Independent Evaluation Group (IEG) reports that only 17% of completed projects included an impact evaluation in their required Implementation Completion and Result Reports (ICRs) [72]. Thus, we suggest that the lack of evaluations for urban slum upgrading is a lost opportunity for improving understanding of what can work for global health. In this final section, we explore practices that might better encourage the political processes and information- gathering needed for measuring the broad health impacts of urban slum upgrading.

Two existing practices, Health Impact Assessment (HIA) and Health in All Policies (HiAP), might help more explicitly incorporate social and environmental determinants of health into urban slum upgrading. Both HIA and HiAP aim to promote rigorous, comprehensive planning processes and to mainstream analyses of health equity throughout public policy-making. HIA seeks to systematically judge the potential (and sometimes unintended) effects of a policy, project, or other intervention upon population health as well as the distribution of those effects. Both a process and a method of analysis, HIA aims for transparent decision-making, integrating multiple health determinants, and assessing the short- and long-term health impacts upon specific groups and general population health [73]. Some local and national governments have formally institutionalized HIA as a distinct practice in public health and other agencies; other governments integrate HIA into existing health analyses and public decision-making analyses. However, to our knowledge, there is no instance where HIA has been used in slum upgrading.

HIA is also a strategy for implementing HiAP, a second integrated approach to decision-making that holds promise for slum upgrading [74]. HiAP recognizes that most public policies can influence health and health equity (either positively or negatively), but policymakers beyond the health sector may not routinely consider these consequences and thus miss opportunities to advance health equity [75]. The impetus for HiAP emerged, in part, from the WHO’s Alma-Ata Declaration in 1978 and reflects numerous international calls for policy action to address the social determinants of health. The European Union and numerous governments globally have endorsed HiAP, but we have not seen it applied to either international aid or within national policies to support slum upgrading [76].

Both HIA and HiAP are rooted in relational analyses of place and population health, a framework that may help further elucidate how slum upgrading projects can be designed and evaluated for their potential health impacts [77,78]. For example, in the “relational” view of place and health, interventions aim to avoid the use of traditional, single exposure, disease or risk factor focused strategies and instead seek co-benefits of mutually reinforcing interventions. Similarly, in a relational view, a set of integrated strategies for slum upgrading would need to be implemented at multiple scales, from the national to the local, to have widespread population health impacts. As we discovered in our review, there are slum upgrading projects and policies that aim to embody the relational approach. The examples from Pakistani and Thai cities, as noted above, suggest that a relational approach to urban slum upgrading is not only possible, but can deliver tangible health gains to the urban poor.

A relational approach also demands multiple sources of data to inform project design, implementation and evaluation. For example, oral histories that capture local meanings and a “sense of place”, are combined with quantitative measures of exposures and geography in a relational view of place. Again, we found that some integrated slum upgrading projects in Brazil, among others, valued resident experiences, knowledge and oral histories, along-side other forms of measurement data.

A relational approach to upgrading would also analyze the shifts, if any, in governance (rules, norms, laws and institutional procedures or practices) that often create slums and might act to stymie opportunities for the urban poor to make healthy decisions [4,5,9]. Thus, a relational approach would examine any changes in power between slum-dwellers and other institutions, particularly the state. The projects we reviewed rarely explored these types of governance impacts, although studies in Brazil, Colombia and Thailand did discuss changes in state-community relations. In Table 3, we detail the potential pathways between slum upgrading and key determinants of health equity.

## 6. Conclusions

Urban slum upgrading is a process and set of outcomes that can positively influence multiple determinants of health and potentially reduce health inequities experienced by the urban poor. The review presented here suggests that more work is needed to integrate clear social determinants criteria into slum upgrading projects’ design and evaluations. When health is included in urban slum upgrading, it is often limited to a single disease, exposure or risk factor, rather than the multiple criteria for health recognized by the WHO findings on the social determinants of health. The global community’s failure to recognize urban slum upgrading as an environmental health equity intervention is a lost opportunity and one that, if it continues, may hinder our ability to meet the Sustainable Development Goals.

The review presented here was limited by focusing only on published evaluations of past slum upgrading projects and policies, all of which were published in English and Spanish. Important projects with only one evaluation and those published in other languages may have been missed. We also acknowledge that slum upgrading projects and related evaluations may be intentionally narrow, in order to respond to financing and/or donor requirements. Development agencies and foundations that typically finance slum upgrading increasingly require quantifiable outputs and might measure a narrow set of health outcomes, rather than the subtler, often qualitative and harder to measure environmental and social determinants discussed here. However, the use of mixed-methods evaluations, such as in-depth interviews, focus group discussions, before-and-after spatial mapping, and longitudinal cohort surveys, could all deepen understanding of how slum upgrading can influence the multiple drivers of human health in cities. Further research can fine-tune these evaluation techniques, and slum upgrading remains an important health promotion strategy urgently requiring the attention of public health practitioners.

## Figures and Tables

**Table 1:** Slum definitions and select health risks.

Example Slum Characteristics	Definition and Indicators (Examples)	Community Health Risks (Select)
Overcrowding	>2 persons/room or <5 m^2^ per person	Spread of TB, influenza, meningitis, skin infections and rheumatic heart disease [16].
Low-Quality Housing Structure	Inferior building materials dirt floors & substandard construction	Vulnerability to floods, extreme heat/cold, burns and falling injuries [17].
Hazardous Housing Sites	Geological and site hazards (e.g., industrial waste sites, garbage dumps, railways, wetlands, steep slopes, etc.)	Acute poisoning; unintentional injuries, landslides, flooding, toxic contamination, environmental pollutants, leptospirosis, cholera, malaria, dengue, hepatitis, drowning [18].
Inadequate Water Access	<50% of households have affordable, 24/7 access to piped water/public standpipe	Malaria, dengue and diarrheal diseases, cholera, typhoid, hepatitis; increased HIV/AIDS vulnerability [19].
Inadequate Sanitation Access	<50% of households with sewer, septic tank, pour-flush or ventilated improved latrine	Fecal-oral diseases, hookworms, roundworm; missed school-days during girls’ menstruation; malnutrition and children’s stunting; safety/sexual violence for women from unsafe toilets [20].
Limited Services and Infrastructure	Inadequate healthcare, drainage, roads, energy, transport, schools, and/or refuse collection	Traffic injuries; lack emergency provision; fires; flooding/drowning; waste burning and air pollution; respiratory diseases and cancer [21].
Tenure Insecurity	Lack of formal title deeds to land and/or structure	Fear; increased hypertension; diabetes; low birth weight newborns [22].
Poverty and Informal Livelihoods	Low incomes, few assets, and access to credit; lack of social protection	Increased occupational hazards; maternal health complications; vaccine-preventable diseases [23]; perinatal diseases; drug-resistant infections;
Violence and Insecurity	Elevated crime, including domestic and gender-based violence	Homicides; hypertension; obesity; sexual violence; vulnerability to STIs, esp for young people forced into sex work [24].
Political Disempowerment	Low or no governmental responsiveness to needs and services	Lack of health services; poor education; preventable hospitalizations; typhus, leptospirosis, cholera, chronic respiratory diseases, growth retardation [25].

**Table 2:** Integrated slum upgrading programs and measured health impacts.

Project Name and Location (Reference)	Focus of Upgrading	Health and Well-Being Impacts (If Measured)
Visakhaptnam, Indore and Vijaywada Upgrading, India [30,31]	Roads, water, lighting, social services and micro-loans	Safety and reduced women’s time burdens
Slum Networking Project (SNP), Ahmedabad, India [32,33,34]	Infrastructure, governance, electrification	Reduction in water-borne illness
SPARC, the National Slum Dwellers Federation and Mahila Milan, Mumbai and Pune, India [35,36]	Water and sanitation, toilets, community governance	None explicitly measured
Baan Mankong, Bangkok, Thailand [37,38]	Housing, tenure, infrastructure, daycare, services for elderly	None explicitly measured
Kampong Improvement Project (KIP), Indonesia [39,40]	Piped water, housing improvements, flood risk reduction	None explicitly measured
Zonal Improvement Program (ZIP), Manila, Philippines [41,42]	Water, roads, housing, land rights, electricity	Reduced incidence diarrhea
Neighborhood Upgrading and Shelter, Indonesia [43,44]	Roads, streetlights, water, toilets, solid waste management	Avoided health costs
Karachi, Orangi Pilot Project (OPP), Karachi, Pakistan [45,46]	Water, sanitation, capacity-building	Reduced Infant Mortality
PRIMED, Medellín, Colombia [47,48]	Housing tenure, physical infrastructure	Improved safety perceptions
Favela Bairro, Rio de Janeiro, Brazil [49,50]	Infrastructure, housing, social programs	Mortality from parasitic or viral vector-born infections; infant mortality; homicides
Bairro Legal, Sao Paulo, Brazil [51,52]	Infrastructure, housing, social and economic development	Improved flood control
Ribeira Azul and Technical and Social Support Project, Salvador, Brazil [53,54]	Infrastructure, housing, social programs	Self-reported reduction crime
Mexico, Piso Firme [55,56]	Cement floors in housing	Children’s parasitic infestations, diarrhea and anemia
PRODEL, Nicaragua [57,58]	Housing, infrastructure, microloans, community savings	None explicitly measured
Citizen Security in Cali, Bogotá, and Medellín, Colombia [59,60]	Social programs, violence prevention	Homicide and inter-personal violence
Huruma Community-Led Upgrading, Nairobi, Kenya [61,62]	Infrastructure, housing, community savings	None explicitly measured
Imizamo Yethu Upgrading, Cape Town, South Africa [63,64]	Housing, water and sanitation infrastructure	None explicitly measured
Hanna Nassif Upgrading, Dar es Salaam, Tanzania [27,65]	Infrastructure, employment, tenure, transport	Reduced waterborne diseases (unspecified)
Kenya Slum Upgrading Program (KENSUP) [66,67,68]	Housing, governance	None specified

**Table 3:** Select upgrading characteristics and related health benefits.

Slum Upgrading Characteristic (Select)	Health Influences (Examples)
Community Empowerment and Political Recognition via Participatory Upgrading	Trust; empowerment; control of life decisions [28]
Right to Remain (In Situ Upgrading)	Social connections; collective efficacy; no fear of displacement [79]
Housing Improvements and Land Tenure	Reduced anxiety from fear of displacement; address can result in social services, access to banking, etc. [80]
Safety and Security	Reduced gender-based violence; reduced physical violence; improved mental health [81]
Integration of Slums into Formal City	Transportation and access to employment, education and services; reduced isolation and segregation [82]
Poverty Reduction	Income for food, electricity and other services [8]
Climate Change Resilience	Reduced health impacts from flooding, heat events, or water scarcity due to drought [83]
